# Supplementary material for: Biomarkers in Liquid Biopsies for Prediction of Early Liver Metastases in Pancreatic Cancer
Source: Cancers (Basel). 2022 Sep 22;14(19):4605. doi: 10.3390/cancers14194605 (PMC9562670; doi:10.3390/cancers14194605)
Supplement: Supplementary file 1 [file cancers-14-04605-s001.zip › Supplementary Figure S1.pdf]

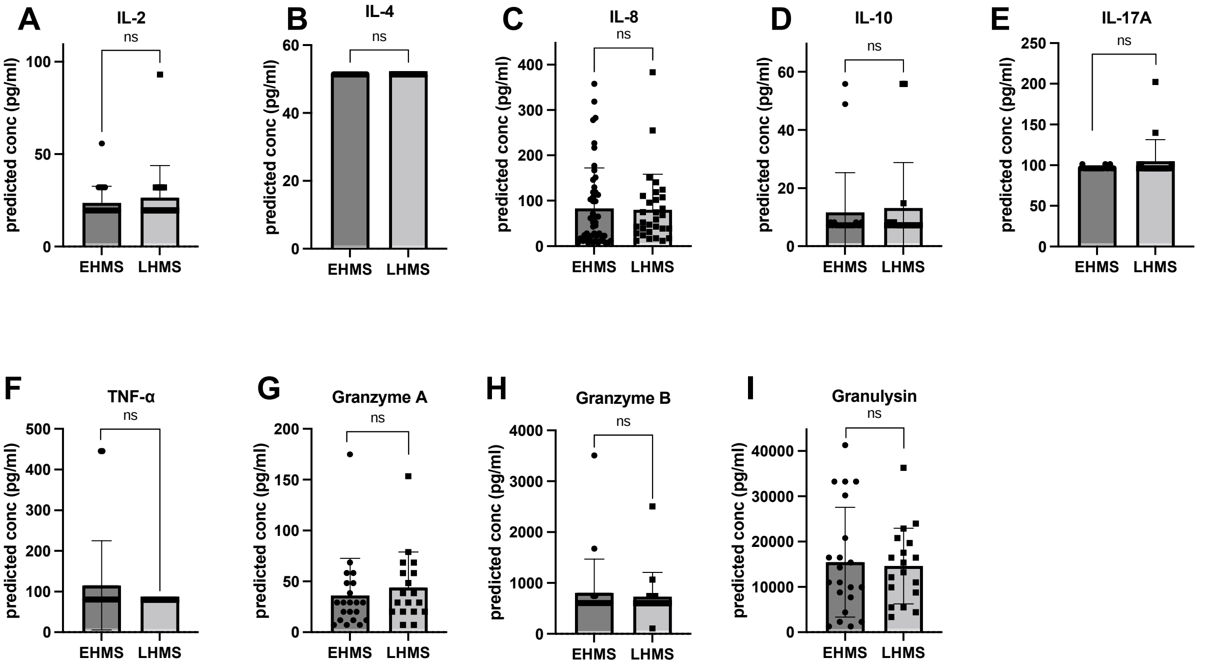

Supplementary Figure S1. Serum levels of inflammatory markers not reaching statistical differences between PDAC-patients with early hepatic metastatic spread (EHMS, ≤ 12 months after surgery) and late hepatic metastatic spread (LHMS, >12 months after surgery) using LEGENDPlex™-analysis. IL-2 (A), IL-4 (B), IL-8 (C), IL-10 (D), IL-17A (E), TNF-α (F), Granzyme A (G), Granzyme B (H) and Granulysin (I). Data are presented as median.
