# Supplementary material for: Biomarkers in Liquid Biopsies for Prediction of Early Liver Metastases in Pancreatic Cancer
Source: Cancers (Basel). 2022 Sep 22;14(19):4605. doi: 10.3390/cancers14194605 (PMC9562670; doi:10.3390/cancers14194605)
Supplement: Supplementary file 1 [file cancers-14-04605-s001.zip › Supplementary Figure S4.pdf]

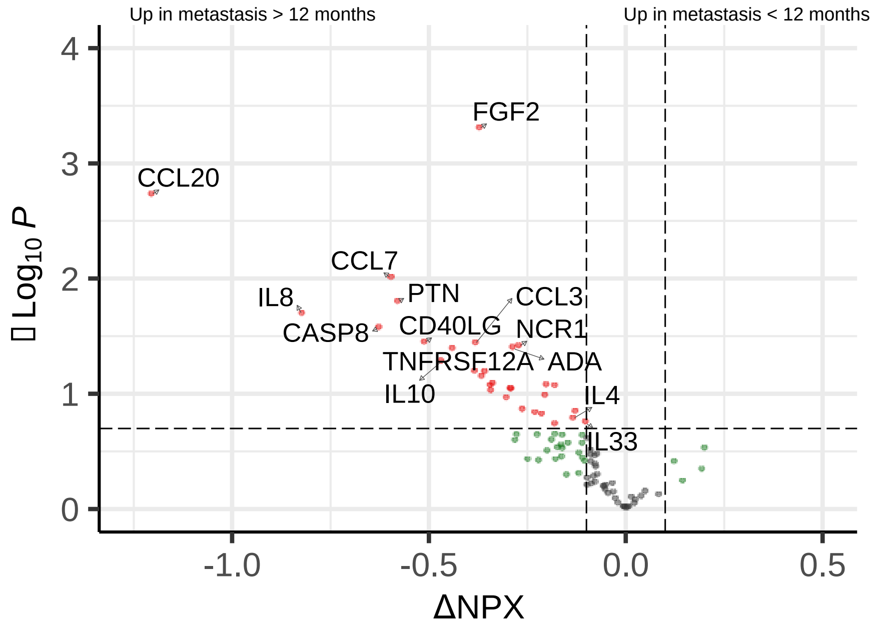

Supplementary Figure S4. Volcano plot of rank product test: Differential protein abundance in PDAC-patients with early hepatic metastatic spread (≤ 12 months) and late hepatic metastatic spread (> 12 months). The x and the y axes depict the NPX differences and the negative log10 of the *p*-value significance.
