# Supplementary material for: Biomarkers in Liquid Biopsies for Prediction of Early Liver Metastases in Pancreatic Cancer
Source: Cancers (Basel). 2022 Sep 22;14(19):4605. doi: 10.3390/cancers14194605 (PMC9562670; doi:10.3390/cancers14194605)
Supplement: Supplementary file 1 [file cancers-14-04605-s001.zip › Supplementary Figure S5.pdf]

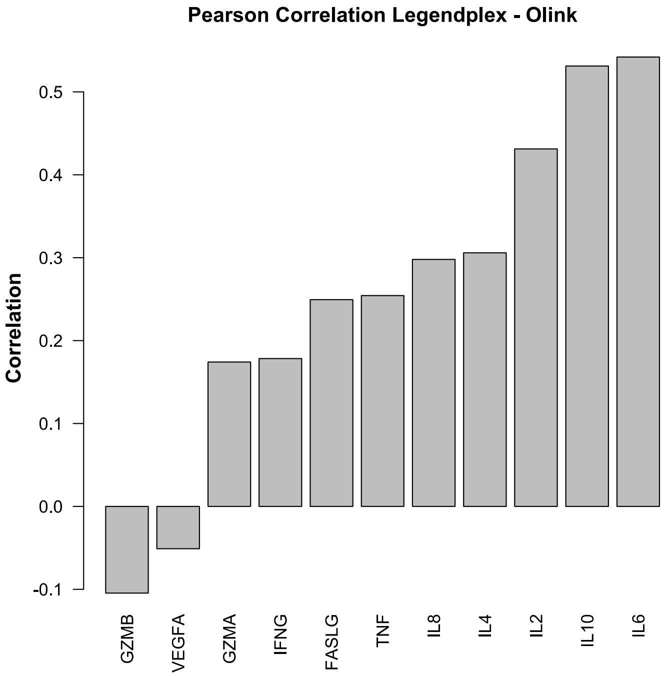

Supplementary Figure S5. Pearson Correlation of LEGENDPlex™- and Olink®-analysis showing good correlation between inflammatory markers analyzed by both methods. FASLG: FasLigand; GZMA: Granzyme A, GZMB: Granzyme B, INFG: Interferon-γ; TNF: Tumor Necrosis Factor; IL2: Interleukin-2; IL4: Interleukin-4; IL6: Interleukin-6; IL8: Interleukin-8; IL10: Interleukin-10; VEGFA Vascular Endothelial Growth Factor A.
