# Supplementary material for: Biomarkers in Liquid Biopsies for Prediction of Early Liver Metastases in Pancreatic Cancer
Source: Cancers (Basel). 2022 Sep 22;14(19):4605. doi: 10.3390/cancers14194605 (PMC9562670; doi:10.3390/cancers14194605)
Supplement: Supplementary file 1 [file cancers-14-04605-s001.zip › Supplementary Figure S6.pdf]

Protein	EHMS	LHMS
FGF2	1,84	2,12
PTN	2,94	3,26
MCP-3	3,42	4,03
NCR1	5,73	5,94
CASP8	6,48	6,9
ADA	6,57	6,69
CCL20	6,6	7,49
TNFRSF12A	6,77	7,17
CCL3	7,25	7,61
IL8	7,55	8,33
CD40LG	8,69	9,12
MCP-4	10,84	11,23
CD40	11,08	11,34

Supplementary Figure S6: Heatmap of the LIMMA-Test results of significantly differentially expressed markers tested by Olink® -analysis in serum of PDAC-patients with EHMS and LHMS. Colour encoded presentation of average expression of respective proteins (green low expression, red: high expression). ADA: Adenosine Deaminase; CASP8: Caspase 8; CCL3: Chemokine ligand 3; CCL20: Chemokine ligand 20; CD40: Cluster of differentiation 40; CD40LG: Cluster of differentiation 40 ligand; FGF2: Fibroblast Growth Factor 2; IL8: interleukin 8; MCP-3/-4: Monocyte chemoattractant protein-3/-4; NCR1: Natural cytotoxicity triggering receptor 1; PTN: Pleiotrophin; TNFRSF12A: Tumor Necrosis Factor Receptor Superfamily Member 12A.
