# Supplementary material for: Biomarkers in Liquid Biopsies for Prediction of Early Liver Metastases in Pancreatic Cancer
Source: Cancers (Basel). 2022 Sep 22;14(19):4605. doi: 10.3390/cancers14194605 (PMC9562670; doi:10.3390/cancers14194605)
Supplement: Supplementary file 1 [file cancers-14-04605-s001.zip › Supplementary Table S1.pdf]

Supplementary Table S1 Demographic data of all PDAC-patients (n=83) included in this study. G: Grade; L: Lymphatic infiltration; N: Nodal infiltration; Pn: Perineural; R= resection; SD: Standard deviation; T: Tumor; UICC: Union international contre le cancer; V: Venous infiltration. In some cases, clinical data are missing.

**Clinical Data**		
Age (years) mean ± SD		65.6 ± 10.2
Sex (males) *n*,%		43, 51.8
**Oncological Data**		
UICC-stage		
	IA (*n*, %)	2, 2.4
	IB (*n*, %)	8, 9.8
	IIA (*n*, %)	18, 22.0
	IIB (*n*, %)	47, 57.3
	III (*n*, %)	2, 2.4
	IV (*n*, %)	
pT	pT1 (*n*, %)	3, 3.7
	pT2 (*n*, %)	24, 29.3
	pT3 (*n*, %)	53, 64.6
	pT4 (*n*, %)	2, 2.4
pN	pN0 (*n*, %)	29, 35.4
	pN1 (*n*, %)	36, 43.9
	pN2 (*n*, %)	17, 20.7
	N infiltrated (*n*, %)	2.9 ± 2.4
	N resected (*n*, %)	22.2 ± 12.2
pL	pL0 (*n*, %)	26, 44.8
	pL1 (*n*, %)	32, 55.2
pV	pV0 (*n*, %)	46, 79.3
	pV1 (*n*, %)	12, 20.7
pPn	pPn0 (*n*, %)	8, 14.5
	pPn1 (*n*, %)	47, 85.5
G	G1 (*n*, %)	1, 1.4
	G2 (*n*, %)	33, 45.2
	G3 (*n*, %)	39, 53.4
R	R0 (*n*, %)	83, 100.0
	R1 (*n*, %)	0, 0.0
**Survival Data**		
Follow up (months ± SD)		11.8 ± 9.1
Survival (months ± SD)		14.5 ± 15.1
Recurrence free survival		11.8 ± 9.0
