# Supplementary material for: Biomarkers in Liquid Biopsies for Prediction of Early Liver Metastases in Pancreatic Cancer
Source: Cancers (Basel). 2022 Sep 22;14(19):4605. doi: 10.3390/cancers14194605 (PMC9562670; doi:10.3390/cancers14194605)
Supplement: Supplementary file 1 [file cancers-14-04605-s001.zip › Supplementary Table S2.pdf]

Supplementary Table S2 Binary logistic regression model for predictors of early (≤12 months) or late (>12 months) hepatic metastatic spread after curative (R0) resection of PDAC. CI: confidence interval (95%); G: Grade; IFN-γ: interferon-γ; IL; Interleukin; L: Lymphatic infiltration; N: Nodal infiltration; OR: Odds ratio; Pn: Perineural infiltration; sFAS: soluble FAS receptor; sFASL: soluble FAS ligand; T: Tumor; TNF-α: Tumor Necrosis Factor-α; UICC: Union international contre le cancer; V: Venous infiltration; VEGF: Vascular Endothelial Growth Factor.

Parameters	Univariate OR (95% CI)	p-value	Multivariate OR (95% CI)	p-value
**Clinical Data**				
Age at surgery *(years)*	1.005 (0.962 – 1.050)	0.837		
Gender *(male vs female)*	1.244 (0.511 – 3.033)	0.630		
**Oncological Data**				
UICC-stage IA *(yes vs no)*	1.759 (0.106 – 29.183)	0.694		
UICC-stage IB *(yes vs no)*	0.529 (0.100 – 2.799)	0.454		
UICC-stage IIA *(yes vs no)*	0.800 (0.266 – 2.403)	0.691		
UICC-stage IIB *(yes vs no)*	1.357 (0.549 – 3.356)	0.509		
UICC-stage III *(yes vs no)*	0.648 (0.118 – 3.562)	0.618		
pT1 *(yes vs no)*	3.517 (0.306 – 40.489)	0.313		
pT2 *(yes vs no)*	0.334 (0.110 – 1.015)	0.053		
pT3 *(yes vs no)*	1.656 (0.638 – 4.295)	0.300		
pT4 *(yes vs no)*	1.700 (0.103 – 28.188)	0.711		
pN0 *(yes vs no)*	0.827 (0.323 – 2.120)	0.693		
pN1 *(yes vs no)*	2.109 (0.853 – 5.213)	0.106		
pN2 *(yes vs no)*	0.291 (0.076 – 1.110)	0.071		
N infiltrated *(n)*	0.938 (0.807–1.090)	0.401		
N resected *(n)*	1.011 (0.972–1.050)	0.593		
pL0 *(yes vs no)*	2.429 (0.835 – 7.006)	0.103		
pL1 *(yes vs no)*	0.412 (0.142 – 1.198)	0.103		
pV0 *(yes vs no)*	4.667 (1.112 – 19.589)	**0.035**	22.905 (1.630 – 321.876)	**0.020**
pV1 *(yes vs no)*	0.762 (0.179 – 3.247)	0.713		
pPn0 *(yes vs no)*	1.739 (0.372 – 8.124)	0.482		
pPn1 *(yes vs no)*	1.273 (0.228 – 7.094)	0.783		
G1 *(yes vs no)*	0.000 (0.000 – 0.000)	1.000		
G2 *(yes vs no)*	1.105 (0.430 – 2.840)	0.835		
G3 *(yes vs no)*	0.920 (0.362 – 2.337)	0.861		
Ca19-9	1.000 (1.000 – 1.000)	0.369		
Granulysin	1.000 (1.000 – 1.000)	0.819		
Granzyme A	0.999 (0.993 – 1.004)	0.623		
Granzyme B	1.000 (1.000 – 1.000)	0.704		
IFN-γ	1.002 (1.000 – 1.000)	0.105		
IL-2	0.993 (0.969 – 1.018)	0.577		
IL-4	0.904 (0.000 – xxx)	1.000		
IL-6	0.984 (0.952 – 1.016)	0.320		
IL-8	1.000 (0.995 – 1.995)	0.925		
IL-10	0.994 (0.968 – 1.021)	0.661		
IL17-A	1.003 (0.980 – 1.026)	0.817		
Perforin	1.000 (1.000 – 1.000)	**0.029**	1.000 (1.000 – 1.000)	**0.049**
sFAS	1.000 (1.000 – 1.000)	0.108		
sFASL	0.864 (xxx-xxx)	0.640		
TNF-α	0.996 (0.986 – 1.005)	0.367		
VEGF	0.998 (0.995 – 1.001)	0.117		
