# Supplementary material for: Biomarkers in Liquid Biopsies for Prediction of Early Liver Metastases in Pancreatic Cancer
Source: Cancers (Basel). 2022 Sep 22;14(19):4605. doi: 10.3390/cancers14194605 (PMC9562670; doi:10.3390/cancers14194605)
Supplement: Supplementary file 1 [file cancers-14-04605-s001.zip › Supplementary Table S3.pdf]

Supplementary Table S3 LIMMA-Test results of markers tested by Olink® -analysis in serum of PDAC-patients with EHMS and LHMS. ADA: Adenosine Deaminase; ADGRG1: Adhesion G-protein couple receptor G1 precursor; ANGPT1: Angiopoietin-1; ANGPT2: Angiopoietin-2; ARG1: Arginase 1; CA19-9: Carbohydrate-Antigen 19-9; CASP8: Caspase 8; CCL3: Chemokine ligand 3; CCL4: Chemokine ligand 4; CCL8: Chemokine ligand 8; CCL17: Chemokine ligand 17; CCL19: Chemokine ligand 19; CCL20: Chemokine ligand 20; CCL23: Chemokine ligand 23; CD: Cluster of differentiation; CD40LG: Cluster of differentiation 40 ligand; CRTAM: Cytotoxic and regulatory t-cell molecule precursor; CSF1: Colony Stimulating Factor 1; CXCL: c-x-c motif chemokine ligand CX3CL1: Recombinant mouse fractalkine protein; DCN: Decorin; EGF: Epithelial Growth Factor; EHMS: Early hepatic metastatic spread; FASLG: FAS ligand; FGF2: Fibroblast Growth Factor 2; GZM: Granzyme; HGF: Hepatocyte Growth Factor; HMOX1: Heme oxygenase 1; ICOSLG: Inducible t-cell co-stimulator ligand; IL-: interleukin IL12RB1: Interleukin 12 receptor beta 1; IFNG: Interferon gamma; KDR: Kinase insert domain receptor; KIR3DL1: Killer cell immunoglobulin-like receptor 3dl1; KLRD1: Killer cell lectin-like receptor subfamily D member 1 LAG3: Lymphocyte-activation gene 3; LAMP3: Lysosome-associated membrane glycoprotein 3; LGALS1: Galactine 1; LGALS9: Galactine 9; LHMS: Late hepatic metastatic spread; MCP: Monocyte chemoattractant protein MICB: MHC class I Polypeptide-Related Sequence B; MMP: Matrix metalloproteinase MUC-16: Mucine 16; NCR1: Natural cytotoxicity triggering receptor 1; NOS3: Nitric oxide synthase 3; PDCD1: Programmed cell death protein 1 precursor; PDCD1LG2: Programmed cell death protein 1 precursor ligand 2; PDGFB: Platelet-derived growth factor subunit B precursor; PD-L1: Programmed death ligand 1; PGF: Placenta Growth Factor; PTN: Pleiotrophin; TGFB1: Transforming Growth Factor Beta 1; TIE2: Tyrosine-protein kinase receptor 2; TNF: Tumor Necrosis Factor; TNFRSF: Tumor Necrosis Factor Receptor Superfamily Member; TRAIL: Tumor Necrosis Factor-related Apoptosis-inducing Ligand; VEGFA: Vascular Endothelial Growth Factor A. *p*-values <0.05 were rated significant and are marked in bold. LogFC, t and adjusted *p*-value denote to the log-scaled fold change in NPX values, the t-statistics and the multiple-testing adjusted *p*-values according to Benjamini-Hochberg, respectively.

Protein	Average Expression EHMS	Average Expression LHMS	logFC	Average Expression	t	*p*-value	adjusted *p*-value
**ADA**	6.57 ± 0.64	6.69 ± 0.52	0.27	6.63	2.09	**0.039**	**0.301**
**ADGRG1**	3.43 ± 0.93	3.70 ± 1.05	0.27	3.52	1.29	0.200	0.558
**ANGPT1**	9.84 ± 0.28	9.92 ± 0.16	0.14	6.03	0.83	0.411	0.630
**ANGPT2**	6.65 ± 0.69	6.60 ± 0.44	0.04	9.87	0.59	0.560	0.710
**ARG1**	5.99 ± 0.76	6.06 ± 0.72	-0.05	6.64	-0.39	0.694	0.809
**CA19-9**	5.34 ± 0.86	5.50 ± 0.84	0.09	5.43	0.48	0.629	0.759
**CASP8**	6.48 ± 1.07	6.90 ± 1.37	0.56	6.63	2.18	**0.032**	0.291
**CCL3**	7.25 ± 0.76	7.61 ± 0.74	0.38	7.36	2.31	**0.023**	0.291
**CCL4**	7.32 ± 0.71	7.69 ± 0.69	0.29	7.44	1.88	0.063	0.368
**CCL8**	8.71 ± 0.74	8.65 ± 0.58	-0.01	8.68	-0.07	0.941	0.962
**CCL17**	10.60 ± 1.17	10.41 ± 0.90	-0.18	10.56	-0.76	0.452	0.670
**CCL19**	11.75 ± 1.10	11.80 ± 1.04	0.20	11.78	0.83	0.411	0.630
**CCL20**	6.60 ± 1.57	7.49 ± 1.73	1.02	7.00	2,83	**0.006**	0.265
**CCL23**	10.49 ± 0.81	10.57 ± 0.53	0.09	10.52	0.55	0.585	0.729
**CD4**	4.14 ± 0.49	4.21 ± 0.36	0.09	4.17	0.98	0.331	0.598
**CD5**	6.13 ± 0.59	6.18 ± 0.36	0.05	6.15	0.44	0.661	0.780
**CD8A**	8.89 ± 0.93	9.23 ± 0.68	0.26	9.07	1.40	0.166	0.526
**CD27**	8.71 ± 0.53	8.68 ± 0.45	0.01	8.70	0.09	0.931	0.962
**CD28**	1.56 ± 0.36	1.61 ± 0.27	0.09	1.58	1.14	0.257	0.593
**CD40**	11.08 ± 0.65	11.34 ± 0.56	0.29	11.15	2.11	**0.038**	0.301
**CD40LG**	8.69 ± 1.21	9.12 ± 0.72	0.56	8.83	2.20	**0.030**	0.291
**CD70**	4.77 ± 0.67	4.56 ± 0.45	-0.15	4.70	-1.11	0.272	0.598
**CD83**	3.18 ± 0.47	3.37 ± 0.51	0.20	3.24	1.86	0.067	0.368
**CD244**	7.04 ± 0.54	7.16 ± 0.41	0.16	7.08	1.46	0.148	0.496
**CRTAM**	6.21 ± 0.86	6.20 ± 0.59	0.14	6.22	0.837	0.405	0.630
**CSF1**	11.11 ± 0.35	11.18 ± 0.31	0.07	11.15	0.92	0.358	0.598
**CXCL1**	10.03 ± 0.73	10.16 ± 0.47	0.14	10.10	0.96	0.340	0.980
**CXCL5**	12.68 ± 0.94	12.88 ± 0.65	0.23	12.74	1.21	0.228	0.589

**CXCL9**	7.47 ± 0.92	7.64 ± 0.67	0.18	7.48	0.95	0.342	0.598
**CXCL10**	9.87 ± 1.10	10.24 ± 0.93	0.23	9.97	0.97	0.334	0.598
**CXCL11**	8.70 ±1.08	8.81 ± 0.82	0.13	8.71	0.60	0.553	0.711
**CXCL12**	1.65 ± 0.39	1.76 ± 0.32	0.10	1.72	1.17	0.246	0.593
**CXCL13**	8,88 ± 0.76	8.91 ± 0.54	0.10	8.91	0.64	0.526	0.691
**CX3CL1**	4.80 ± 0.59	4.82 ± 0.51	0.09	4.82	0.71	0.480	0.680
**DCN**	5.03 ± 0.41	5.08 ± 0.36	0.03	5.08	0.35	0.724	0.822
**EGF**	9.64 ± 0.95	9.92 ± 0.69	0.36	9.71	1.76	0.082	0.368
**FASLG**	6.99 ± 0.55	7.00 ± 0.54	-0.02	7.00	-0.13	0.895	0.957
**FGF2**	1.84 ± 0.41	2.12 ± 0.48	0.39	1.93	4.04	**<0.001**	**0.010**
**GZMA**	7.59 ± 0.60	7.52 ± 0.46	-0.01	7.58	-0.04	0.975	0.967
**GZMB**	3.48 ± 0.67	3.50 ± 0.60	0.09	3.48	0.64	0.524	0.691
**GZMH**	4.74 ± 0.94	4.63 ± 0.72	-0.04	4.67	-0.22	0.826	0.894
**HGF**	10.32 ± 0.77	10.51 ± 0.69	0.29	10.42	1.80	0.075	0.368
**HMOX1**	12.53 ± 0.64	12.68 ± 0.73	0.05	12.59	0.31	0.756	0.849
**ICOSLG**	6.64 ± 0.44	6.67 ± 0.38	0.07	6.65	0.74	0.471	0.677
**IFNG**	7.99 ± 1.28	7.94 ± 0.91	-0.17	7.96	-0.69	0.494	0.6872
**IL-1 alpha**	1.36 ± 0.56	1.45 ± 0.36	0.10	1.38	0.85	0.360	0.630
**IL2**	3.56 ± 0.41	3.58 ± 0.24	0.055	3.56	0.67	0.505	0.687
**IL4**	2.39 ± 0.43	2.49 ± 0.33	0.15	2,41	1.74	0.086	0.368
**IL5**	3.88 ± 0.61	4.03 ± 1.08	0.21	3.92	1.21	0.231	0.589
**IL6**	4.50 ± 1.21	4.21 ± 0.96	-0.16	4.47	-0.54	0.594	0.729
**IL7**	7.15 ± 0.84	7.09 ± 0.78	0.01	7.16	0.08	0.936	0.962
**IL8**	7.55 ± 1.34	8.33 ± 1.64	0.77	7.80	2.43	**0.017**	0.291
**IL10**	4.49 ±1.04	4.87 ± 1.07	0.47	4.63	1.97	0.052	0.342
**IL12**	6.78 ± 0.78	6.98 ± 0.63	0.29	6.84	1.75	0.084	0.368
**IL12RB1**	2.77 ± 0.47	2.81 ± 0.28	0.10	2.78	1.06	0.292	0.598
**IL13**	3.17 ± 0.66	3.43 ± 1.11	0.27	3.25	1.45	0.149	0.496
**IL15**	6.53 ± 0.54	6.60 ± 0.47	0.06	6.58	0.48	0.635	0.759
**IL18**	9,42 ± 0.88	9.55 ± 0.89	0.21	9.48	1.10	0.273	0.598
**IL33**	1.28 ± 0.33	1.31 ± 0.28	0.13	1.29	1.78	0.079	0.369
**KDR**	9.29 ± 0.35	9.33 ± 0.34	0.02	9.31	0.26	0.792	0.870
**KIR3DL1**	3.53 ± 0.61	3.63 ± 0.70	0.17	3.55	1.24	0.271	0.587
**KLRD1**	6.70 ± 0.82	6.81 ± 0.53	0.17	6.75	1.05	0.295	0.598
**LAG3**	5.62 ± 0.63	5.76 ± 0.49	0.12	5.65	0.93	0.350	0.598
**LAMP3**	5.82 ± 0.87	5.48 ± 0.72	-0.267	5.70	-1.36	0.178	0.527
**LGALS1**	7.92 ± 0.35	7.95 ± 0.31	0.09	7.92	1.14	0.258	0.593
**LGALS9**	8.76 ± 0.44	8.86 ± 0.44	0.13	8.80	1.29	0.199	0.558
**MCP-1**	11.74 ± 0.64	11.96 ± 0.69	0.17	11.84	1.19	0.254	0.593
**MCP-3**	3.42 ± 0.83	4.03 ± 1.10	0.49	3.67	2.29	**0.024**	0.291
**MCP-4**	10.84 ± 0.93	11.23 ± 0.70	0.38	10.98	2.029	**0.045**	0.321
**MICB**	6.51 ± 0.96	6.46 ± 1.60	0.18	6.52	0.67	0.589	0.687
**MMP7**	13.42 ± 0.50	13.50 ± 0.61	0.10	13.45	0.83	0.409	0.630
**MMP12**	8.12 ± 0.84	8.31 ± 0.92	0.30	8.18	1.58	0.117	0.468
**MUC-16**	3.98 ±1.14	3.86 ± 0.71	-0.06	4.02	-0.26	0.795	0.871
**NCR1**	5.73 ± 0.53	5.94 ± 0.55	0.26	5.82	2.23	**0.028**	0.291
**NOS3**	3.77 ± 0.37	3.80 ± 0.43	0.08	3.77	0.94	0.351	0.598
**PDCD1**	6.26 ± 0.62	6.31 ± 0.51	0.10	6.29	0.78	0.440	0.663
**PDCD1LG2**	3.90 ± 0.42	3.96 ± 0.30	0.13	3.93	1.46	0.148	0.496
**PGF**	8.69 ± 0.52	8.75 ± 0.45	0.11	8.73	0.96	0.340	0.598
**PDGFB**	10.86 ± 0.17	10.84 ± 0.25	-0.04	10.86	-0.72	0.471	0.677
**PD-L1**	7.31 ± 0.65	7.30 ± 0.45	0.07	7.33	0.53	0.594	0.729
**PTN**	2.94 ± 1.02	3.26 ± 1.17	0.61	3.04	2.69	**0.009**	0.265

**TGFB1**	10.45 ± 0.82	10.67 ± 0.39	0.21	10.54	1.38	0.171	0.526
**TIE2**	8.86 ± 0.44	8.96 ± 0.36	0.09	8.92	0.93	0.356	0.598
**TNF**	4.98 ± 0.57	5.19 ± 0.45	0.17	5.00	1.45	0.151	0.496
**TNFRSF4**	6.99 ± 0.63	7.07 ± 0.64	0.15	7.02	1.04	0.302	0.598
**TNFRSF9**	7.32 ± 0.59	7.43 ± 0.53	0.19	7.35	1.47	0.146	0.496
**TNFRSF12A**	6.77 ± 0.85	7.17 ± 0.96	0.47	6.91	2.44	**0.017**	0.291
**TNFRSF21**	7.97 ± 0.38	8.03 ± 0.31	0.08	8.01	0.99	0.327	0.598
**TNFSF12**	8.39 ± 0.55	8.37 ± 0.49	0.01	8.40	0.05	0.957	0.967
**TNFSF14**	7.18 ± 0.96	7.41 ± 0.75	0.34	7.27	1.72	0.088	0.368
**TRAIL**	8.09 ± 0.47	8.20 ± 0.37	0.04	8.13	0.364	0.716	0.822
**VEGFA**	10.63 ± 0.90	10.56 ± 0.60	-0.02	10.64	-0.09	0.928	0.962
