# Supplementary material for: Biomarkers in Liquid Biopsies for Prediction of Early Liver Metastases in Pancreatic Cancer
Source: Cancers (Basel). 2022 Sep 22;14(19):4605. doi: 10.3390/cancers14194605 (PMC9562670; doi:10.3390/cancers14194605)
Supplement: Supplementary file 1 [file cancers-14-04605-s001.zip › Supplementary Table S4.pdf]

Supplementary Table S4 Binary logistic regression model for predictors of early (≤12 months) or late (>12 months) hepatic metastatic spread after curative (R0) resection of PDAC using Olink®-analysis. ADA: Adenosine Deaminase; CASP8: Caspase 8; CCL3: Chemokine ligand 3; CCL20: Chemokine ligand 20; CD40: Cluster of differentiation 40; CD40LG: Cluster of differentiation 40 ligand; CI: Confidence interval; FGF2: Fibroblast Growth Factor 2; IL8: Interleukin-8; MCP-3: Monocyte Chemoattractant Protein-3; MCP-4: Monocyte Chemoattractant Protein-4; NCR1: Natural Cytotoxicity triggering Receptor 1; OR: Odds ratio; PTN: Pleiotrophin; TNFRSF12A: Tumor Necrosis Factor Receptor Superfamily Member 12A. *p*-values <0.05 were rated significant and are marked in bold.

**Parameters**	**Univariate OR (95% CI)**	***p*-value**	**Multivariate OR (95% CI)**	***p*-value**
		**Clinical Data**		
Age at surgery *(years)*	1.154 (0.443 – 3.007)	0.770		
Gender *(male vs female)*	1.003 (0.958 – 1.051)	0.899		
		**Oncological Data**		
UICC-stage IA *(yes vs no)*	1.577 (0.094 – 26.324)	0.751		
UICC-stage IB *(yes vs no)*	0.592 (0.106 – 3.295)	0.549		
UICC-stage IIA *(yes vs no)*	0.640 (0.195 – 2.105)	0.463		
UICC-stage IIB *(yes vs no)*	1.700 (0.633 – 4.566)	0.292		
UICC-stage III *(yes vs no)*	0.760 (0.129– 4.466)	0.761		
pT1 (*yes vs no)*	3.280 (0.283 – 38.064)	0.342		
pT2 (*yes vs no)*	0.320 (0.102 – 1.006)	0.051		
pT3 *(yes vs no)*	1.962 (0.704 – 5.471)	0.198		
pT4 *(yes vs no)*	1.577 (0.094 – 26.324)	0.751		
pN0 *(yes vs no)*	0.735 (0.268 – 2.019)	0.551		
pN1 *(yes vs no)*	2.500 (0.925 – 6.754)	0.071		
pN2 *(yes vs no)*	0.352 (0.088 – 1.405)	0.139		
pL0 *(yes vs no)*	0.735 (0.224 – 2.413)	0.611		
pV0 *(yes vs no)*	0.551 (0.101 – 3.013)	0.492		
pPn0 *(yes vs no)*	1.204 (0.205 – 7.055)	0.837		
G1 *(yes vs no)*	0.667 (0.000 – 0.000)	0.097		
G2 *(yes vs no)*	1.240 (0.442 – 3.476)	0.682		
G3 *(yes vs no)*	0.900 (0.324 – 2.502)	0.840		
ADA	2.179 (0.923 – 5.143)	0.075		
ADGRG1	1.317 (0.810 – 2.139)	0.267		
ANGPT1	2.2577 (0.321 – 20.668)	0.373		
ANGPT2	0.996 (0.466 – 2.137)	0.992		
ARG1	1.152 (0.600 – 2.213)	0.670		
CA19-9	1.241 (0.707 – 2.177)	0.453		
CASP8	1.373 (0.928 – 2.032)	0.113		
CCL3	2.059 (1.064 – 3.985)	**0.032**	0.362 (0.061 – 2.158)	0.264
CCL4	2.517 (1.196 – 5.296)	**0.015**	2.738 (0.528 – 14.206)	0.231
CCL8	1.117 (0.567 – 2.199)	0.750		
CCL17	0.989 (0.634 – 1.544)	0.962		
CCL19	1.202 (0.779 – 1.855)	0.406		
CCL20	1.478 (1.090 – 2.005)	**0.012**	1.185 (0.732 – 1.918)	0.490
CCL23	1.342 (0.679 – 2.651)	0.397		
CD4	1.598 (0.538 – 4.750)	0.299		
CD5	1.299 (0.552 – 3.543)	0.479		
CD8A	1.516 (0.823 – 2.791)	0.218		
CD27	1.186 (0.449 – 3.134)	0.731		
CD28	1.802 (0.432 – 7.514)	0.419		
CD40	2.114 (0.924 – 4.834)	0.076		

CD40LG	1.646 (0.970 – 2.792)	0.065		
CD70	0.760 (0.334 – 1.730)	0.513		
CD83	2.427 (0.882 - 6.683)	0.086		
CD244	1.520 (0.588 – 3.933)	0.380		
CRTAM	1.203 (0.644 – 2.248)	0.562		
CSF1	2.3646 (0.584 – 11.988)	0.207		
CXCL1	1.531 (0.699 – 3.351)	0.287		
CXCL5	1.319 (0.710 – 2.450)	0.348		
CXCL9	1.277 (0.760 – 2.493)	0.329		
CXCL10	1.298 (0.822 – 2.049)	0.262		
CXCL11	1.151 (0.716 – 1.851)	0.561		
CXCL12	2.819 (0.924 – 15.785)	0.064		
CXCL13	1.347 (0.655 – 2.770)	0.418		
CX3CL1	1.304 (0.557 – 3.050)	0.541		
DCN	1.862 (0.522 – 6.643)	0.338		
EGF	1.594 (0.870 – 2.920)	0.132		
FASLG	0.938 (0.394 – 2.232)	0.884		
FGF2	5.712 (1.672 –19.507)	**0.005**	3.221 (0.593 –17.498)	0.176
GZMA	1.002 (0.421 – 2.380)	0.997		
GZMB	1.269 (0.590 – 2.729)	0.542		
GZMH	0.925 (0.531 – 1.612)	0.784		
HGF	1.852 (0.938 – 3.657)	0.076		
HMOX1	1.318 (0.655 – 2.653)	0.440		
ICOSLG	1.377 (0.456 – 4.158)	0.570		
IL-1 alpha	1.170 (0.467 – 2.935)	0.738		
IL2	1.1060 (0.253 – 4.438)	0.937		
IL4	2.113 (0.591 – 7.561)	0.250		
IL5	1.224 (0.685 – 2.186)	0.495		
IL6	0.862 (0.559 – 1.328)	0.499		
IL7	0.978 (0.545 – 1.755)	0.941		
IL8	1.430 (1.021 – 2.003)	**0.037**	1.082 (0.598 – 1.957)	0.793
IL10	1.603 (0.990 – 2.595)	0.055		
IL12	1.958 (0.950 – 4.036)	0.069		
IL12RB1	1.477 (0.468 – 4.658)	0.506		
IL13	1.490 (0.825 – 2.691)	0.186		
IL15	1.795 (0.652 – 4.942)	0.258		
IL18	1.331 (0.781 – 2.269)	0.294		
IL33	1.8503 (0.315 – 10.325)	0.508		
IFNG	1.032 (0.626 – 1.701)	0.901		
KDR	1.382 (0.347 – 5.499)	0.646		
KIR3DL1	1.361 (0.668 – 2.775)	0.396		
KLRD1	1.271 (0.655 – 2.464)	0.479		
LAG3	1.340 (0.594 – 3.023)	0.481		
LAMP3	0.714 (0.405 – 1.258)	0.244		
LGALS1	1.872 (0.440 – 7.964)	0.396		
LGALS9	2.067 (0.699 – 6.118)	0.190		
MCP-1	1.720 (0.824 – 3.590)	0.149		
MCP-3	1.809 (1.070 – 3.057)	**0.027**	1.005 (0.451 – 2.243)	0.990
MCP-4	1.900 (1.006 – 3.586)	**0.048**	1.097 (0.431 – 2.788)	0.847
MICB	1.117 (0.762 – 1.636)	0.570		
MMP7	1.274 (0.518 – 3.131)	0.598		
MMP12	1.410 (0.813 – 2.446)	0.221		

MUC-16	0.938 (0.571 – 1.540)	0.799		
NCR1	2.096 (0.856 – 5.134)	0.105		
NOS3	1.523 (0.441 – 5.259)	0.506		
PDCD1	1.348 (0.606 – 2.999)	0.464		
PDCD1LG2	2.039 (0.569 – 7.302)	0.274		
PGF	1.825 (0.668 – 4.983)	0.241		
PDGFB	0.549 (0.055 – 5.440)	0.608		
PD-L1	1.491 (0.610 – 3.644)	0.381		
PTN	1.704 (1.027 – 2.830)	**0.039**	1.322 (0.719 – 2.431)	0.369
TGFB1	1.757 (0.801 – 3.853)	0.160		
TIE2	1.604 (0.516 – 4.982)	0.414		
TNF	2.477 (0.949 – 6.463)	0.064		
TNFRSF4	1.438 (0.691 – 2.994)	0.331		
TNFRSF9	1.854 (0.763 – 4.500)	0.173		
TNFRSF12A	1.626 (0.949 – 2.788)	0.077		
TNFRSF21	2.238 (0.534 – 9.389)	0.271		
TNFSF12	1.073 (0.448 – 2.570)	0.874		
TNFSF14	1.561 (0.892 – 2.731)	0.119		
TRAIL	1.533 (0.508 – 4.624)	0.448		
VEGFA	1.004 (0.561 – 1.798)	0.990		
