# Supplementary material for: Biomarkers in Liquid Biopsies for Prediction of Early Liver Metastases in Pancreatic Cancer
Source: Cancers (Basel). 2022 Sep 22;14(19):4605. doi: 10.3390/cancers14194605 (PMC9562670; doi:10.3390/cancers14194605)
Supplement: Supplementary file 1 [file Supplementary Figure S2.pdf › Supplementary Figure S2.pdf]

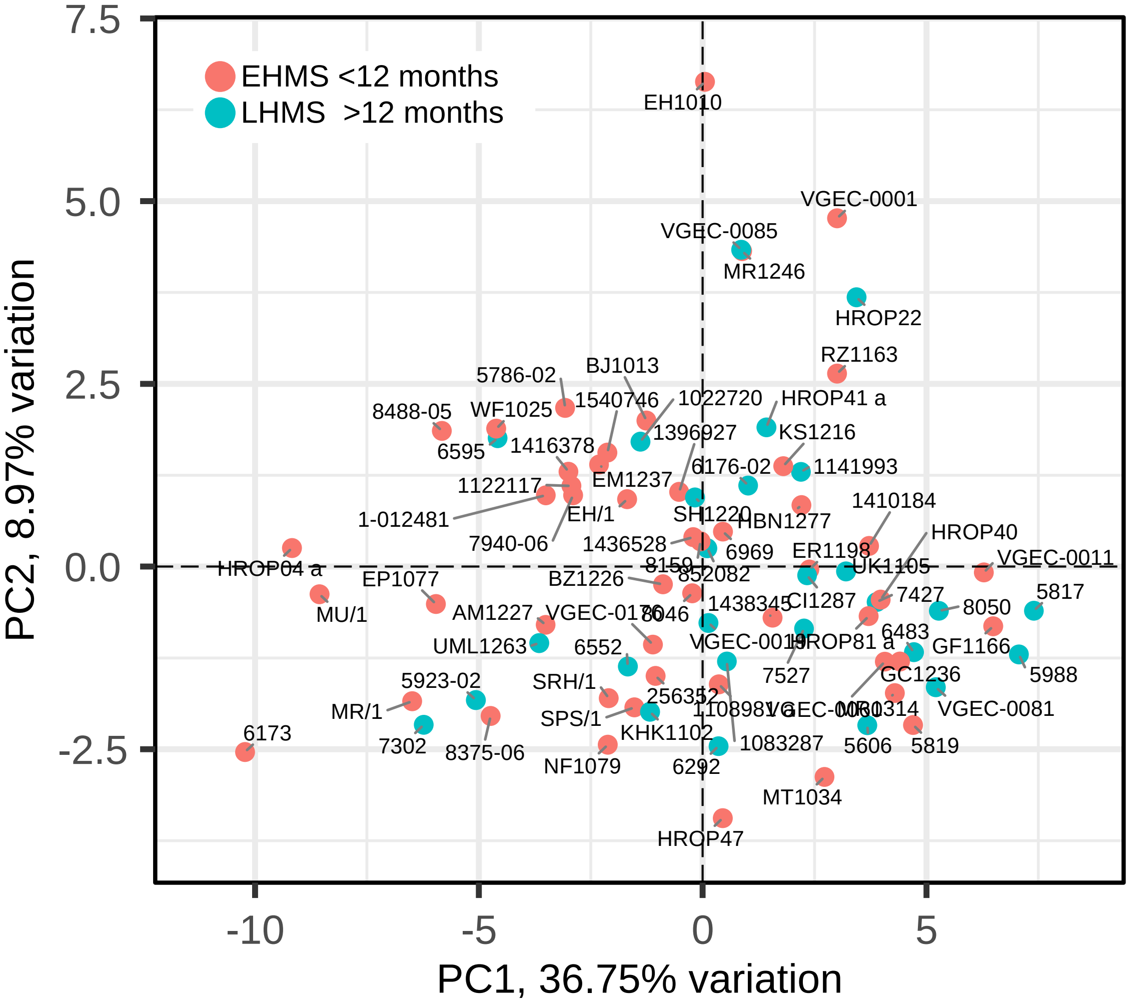

Supplementary Figure S2. Principal component analysis of determined analytes in serum of PDAC-patients with EHMS (≤ 12 months) compared to PDAC-patients with LHMS (> 12 months) using Olink®-analysis. Patient samples do not cluster according to EHMS/LHMS but rather according to the mean NPX values of the protein panels (cf. Suppl. Fig. 3).
