# Supplementary material for: Biomarkers in Liquid Biopsies for Prediction of Early Liver Metastases in Pancreatic Cancer
Source: Cancers (Basel). 2022 Sep 22;14(19):4605. doi: 10.3390/cancers14194605 (PMC9562670; doi:10.3390/cancers14194605)
Supplement: Supplementary file 1 [file Supplementary Figure S3.pdf › Supplementary Figure S3.pdf]

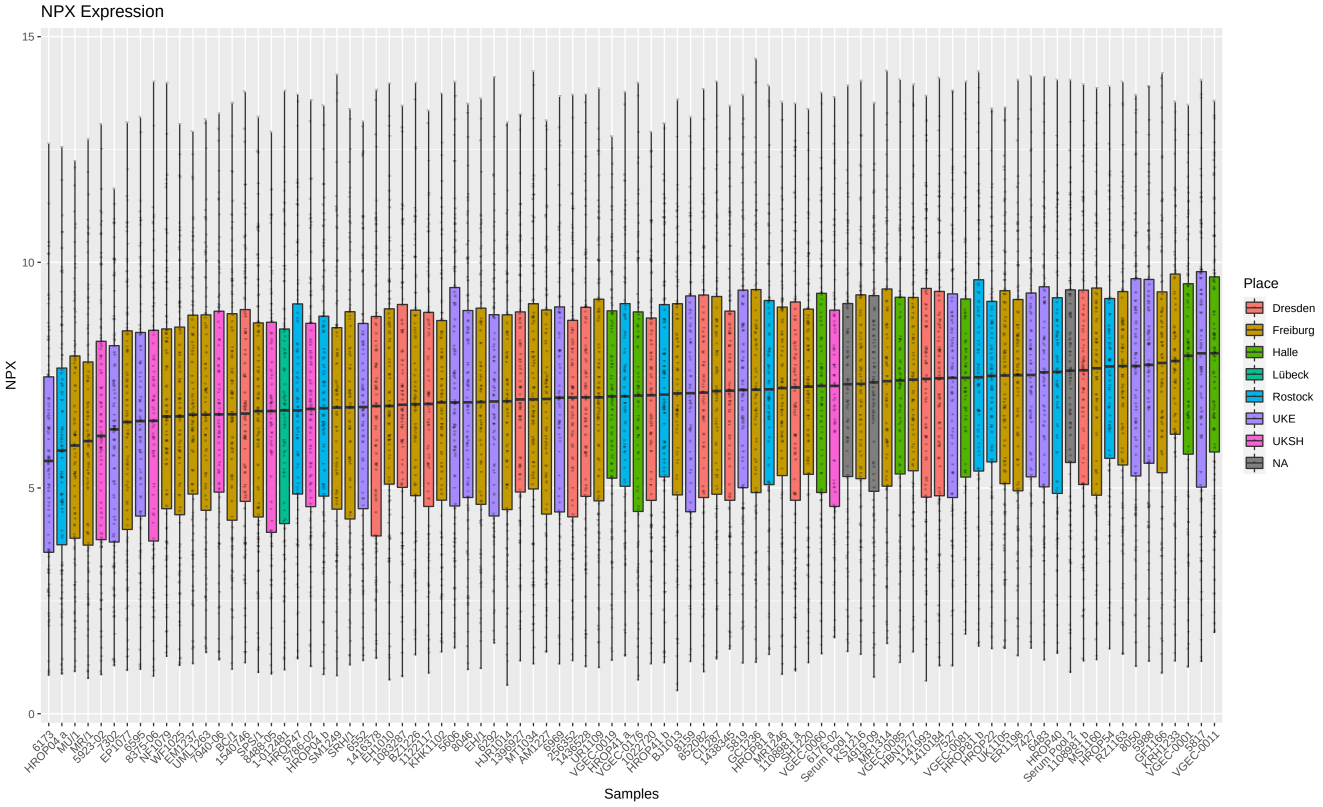

Supplementary Figure S3. Barplot of all analytes determined in serum samples from biobanks of seven board-certified German tertiary referral centers for PDAC ordered by mean NPX values per patient. A linear regression on the mean protein abundance shows a non-zero, positive slope (*p*-value < 2e-16). NPX: normalized protein expression.
